# Differential microvascular endothelial cell responses in the retina in diabetes compared to the heart and kidneys, a spatial transcriptomic analysis

**DOI:** 10.1371/journal.pone.0310949

**Published:** 2024-12-31

**Authors:** Eric Wang, Biao Feng, Shali Chen, Zhaoliang Su, Subrata Chakrabarti

**Affiliations:** 1 Department of Pathology and Laboratory Medicine, Western University, London, Ontario, Canada; 2 International Genome Center, Jiangsu University, Zhenjiang, China; Cedars-Sinai Medical Center, UNITED STATES OF AMERICA

## Abstract

Endothelial cells and high glucose-induced endothelial dysfunction are the common origin of chronic diabetic complications such as retinopathy, nephropathy, and cardiomyopathy. Yet their common origins, the vascular manifestations of such complications are different. We examined the basal heterogeneity between microvascular endothelial cells(MECs) from the retina, kidneys, and heart, as well as their differential responses to hyperglycemia in diabetes. To this extent, we used a spatial transcriptomic approach to investigate gene expression differences across retinal, renal, and cardiac MECs in diabetic and non-diabetic mouse models. We validated MEC heterogeneity *in vitro* using human retinal and cardiac MECs. The spatial transcriptomic approach was also used to explore potential similarities in retinal MECs and neuronal cells in response to hyperglycemia. We found that MECs from different target organs of major diabetic complications were transcriptomically distinct at the basal state and respond differently to hyperglycemia. These findings were recapitulated in cell culture, with selected analytes. We found minimal similarities between retinal MECs and neuronal cells. Our findings show considerable heterogeneity across retinal, renal, and cardiac MECs, both at the basal state and in their responses to hyperglycemia in diabetes. These findings show that organ specific MEC heterogeneity can influence differential development of pathological changes across various target organs of chronic diabetic complications, and suggest that MEC heterogeneity may influence treatment target(s) and drug development.

## Introduction

Diabetes mellitus and its complications are significant causes of mortality, morbidity, and reduced in quality of life [1, 2]. Diabetes is characterized by dysregulation of glucose homeostasis and sustained hyperglycemia, leading to endothelial dysfunction, tissue damage, and the development of chronic diabetic complications [3–5]. Major complications of diabetes include diabetic cardiomyopathy(DCM; a significant risk factor for heart failure), diabetic nephropathy(DN; a leading cause of end stage renal disease and kidney failure), and diabetic retinopathy(DR; the leading cause of vision impairment in working-aged adults) [3–5]. Endothelial dysfunction is an important early component of chronic diabetic complications because endothelial cells(ECs) are particularly susceptible to hyperglycemic damage [6–8]. Endothelial dysfunction leads to vascular dysfunction, which paves the way for further damage to target tissues. Initial glucose-induced endothelial damage has been well characterized and is understood to be common across complications, however glucose-induced endothelial dysfunction may manifest differently in different complications.

DR, the ophthalmic manifestation of chronic diabetes, is the most common chronic microvascular diabetic complication [9]. The retina is an intricate and sensitive tissue, comprising photoreceptors and an integrated grouping of neuronal(amacrine cells, bipolar cells, ganglion cells, and horizontal cells), glial(astrocytes and Müller glia), immune(microglia), and vascular cells(ECs and pericytes) known as the neurovascular unit [10]. The retina is subject to strict homeostatic control through the blood-retinal barrier(BRB), which dynamically regulates blood flow and substrate permeability in response to the metabolic demands of the retina [11–13]. Loss of BRB integrity can lead to disruptions to the retinal homeostasis and cause pathological changes in the retina [11–13]. As is the case with other complications, hyperglycemia-induced endothelial and vascular dysfunction are among the earliest developments DR. DCM and DN, despite also being chronic microvascular disorders of diabetes mediated by hyperglycemia-induced endothelial dysfunction, have limited similarities to DR and to each other. DCM and DN involve fibrosis(partially caused by endothelial dysfunction through endothelial-to-mesenchymal transition), which is not a major feature of DR [3–5]. Increased vascular permeability occurs in all three complications, but is more central to the disease processes of DR and DN [3–5]. Differential vascular presentations between different microvascular diabetic complications may be attributable to EC heterogeneity.

ECs are ubiquitous throughout the body, being found in nearly every organ, and covering a net area of up to 6000 m^2^ [14]. ECs have important roles in both physiological and pathological processes [14, 15]. Despite their ubiquity, ECs exhibit considerable heterogeneity; microvascular ECs(MECs) have different properties to macrovascular ECs [14]. MECs, the centerpieces of microvascular diabetic complications, have functional variations across different tissues. MECs of the neurovascular unit of the retina form the BRB, the highly selective barrier that restricts the free exchange of solutes between the blood and the tissue in order to maintain tight homeostatic control and protect the sensitive neuronal tissues [11–13]. Glomerular MECs of the kidneys on the other hand, form a fenestrated filtration barrier which allow free diffusion of solutes, preventing only proteins and other large solutes from crossing [14, 15]. The cardiac MECs which supply the myocardium form continuous capillaries, the characteristics of which lie somewhere between those of the retina than those of the kidneys [14, 15].

Innate functional differences in MECs may contribute to differential responses to hyperglycemia in organs affected by chronic diabetic complications. Additionally, MECs of the retina, heart, and kidneys are surround by different populations of cells which may create differential environments for each MEC type, further leading to unique responses to hyperglycemic stimuli. We hypothesized that unique functional properties and tissue contexts cause MECs of the heart, kidneys, and retina to exhibit basal transcriptomic differences, i.e. in the non-diabetic condition. We further hypothesized that cardiac, renal, and retinal MECs respond differently to hyperglycemia, resulting in differential manifestations of endothelial damage and dysfunction in chronic diabetic complications. We used a mouse model of diabetes to explore differences in basal MEC transcriptomic profiles and differential responses to hyperglycemia, this can inform MEC heterogeneity in patients with diabetes.

## Methods

### Animals

Animals were cared for according to the Guiding Principles in the Care and Use of Animals. Experimental protocols were approved by Western University Ethics Committee and Animal Care and Veterinary Services. Experiments conform to the Guide for the Care and Use of Laboratory Animals published by the US National Institutes of Health(NIH Publication No. 85–23, revised 1996).

Six C57BL/6J mice were randomly assigned to diabetic and non-diabetic groups(3 each). Diabetes was induced at 8 weeks of age via consecutive intraperitoneal injections of streptozotocin over 5 days(50 mg/kg in citrate buffer, pH 4.5), and diabetes was confirmed via glucometer measurements of over 16.7 mmol/L following the final injection. Non-diabetic littermates were given injections of citrate buffer only. Only male mice were used, because previous work done in our lab using the present model of early diabetes showed no sex differences in organ dysfunction [16, 17]. Mice were kept in a temperature and humidity-controlled facility with environmental enrichment, and with food and water available ad libitum. They were monitored for body weight, blood glucose on a weekly basis over a period of 2 months. Urine ketone and glucose were also monitored semi-quantitatively using urine test strips (URiSCAN GluKeto2, YD Diagnostics Corp). Insulin was available for intervention if blood glucose exceeded reasonable levels. At the end of the second month, mice were euthanized via isoflurane overdose, exsanguination was used as a secondary euthanasia method. We have previously shown that early functional and pathological changes develop within this time span[cite [16–19]]. Eyes, kidneys, and hearts were collected, fixed in 10% neutral-buffered formalin, embedded in paraffin, and sectioned at 5 μm for Nanostring Spatial Transcriptomic Analysis.

### Nanostring digital spatial profiling of mouse transcriptome

**Fig 1:**
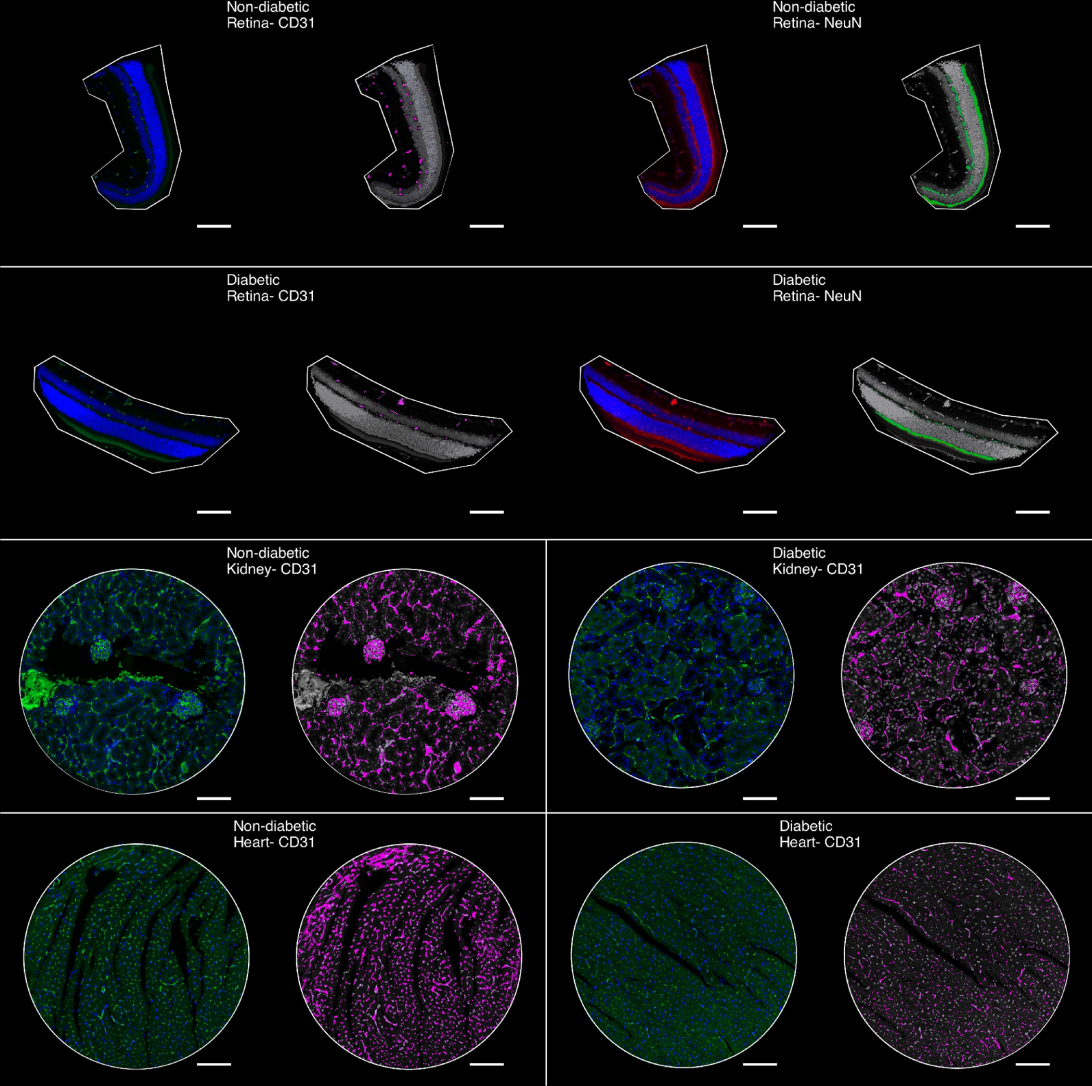
Regions of interest. Representative images of regions of interest from non-diabetic and non-diabetic retinas(A, B), kidneys(C, D), and hearts(E, F). CD31 was stained with TexasRed and pseudo-colored green, while NeuN was stained with Cy5 and pseudo-coloured red for better visual representation. CD31+ and NeuN+ regions were highlighted in magenta and green respectively. Specific sites representing cells of interest were chosen for subsequent UV cleavage and digital spatial analysis. n = 3 per condition/organ, white bar = 100 μm.

Tissue processing and data collection were done in collaboration with Nanostring. Briefly, sections were immunohistochemically stained for a structural endothelial marker, CD31, and hybridized with Mouse Whole Transcriptome Atlas probes(mouse-specific *in situ* hybridization probes cross-linked to unique UV-cleavable barcode sequences). Sections were fluorescently imaged, CD31^+^ cells were digitally highlighted, and regions of interest representing MECs(as opposed to ECs of large vessels) were chosen as regions of interest (Fig 1, S1 Fig). Barcoded oligonucleotides were cleaved from MECs in the regions of interest using focused UV light, retrieved, sequenced, and quantified. Retinal sections were also immunohistochemically stained for the neuronal marker NeuN, and barcoded oligonucleotides were cleaved from NeuN^+^ regions.

Raw data were processed using within GeoMx DSP suite. Initial quality control and biological probe quality control were done using default parameters. 1799 out of 20175 genes were flagged with warnings and were excluded from the remainder of analyses. The remaining 18376 genes were normalized to the 75^th^ percentile and exported for analysis.

### T-test and volcano plots

T-tests were performed to generate volcano plots. Student’s T-test, fold change calculations, and volcano plots were performed and/or generated in Microsoft Excel. Lines representing appropriate cut-offs(p < 0.05, FC > 1.5) were added.

### Pathway analysis

Gene set enrichment analysis was performed using GSEA(version 4.2.1) [20, 21]. “Hallmark” and “Canonical Pathways” gene set collections were analyzed. Gene set enrichment analyses were performed using default parameters save for permutation type which was changed to “gene_set” to better accommodate our sample sizes, as recommended by the manual. Gene sets which were enriched at p < 0.05 and q < 0.25 were deemed significant in this exploratory analysis.

### Network visualization and clustering

Visualization and grouping of differentially enriched pathways(gene sets) was performed using Cytoscape [22] (version 3.10.1) using the EnrichmentMap Pipeline Collection comprising EnrichmentMap, clusterMaker2, WordCloud, and AutoAnnotate, according to their manual [23].

### Dimension reduction analysis

Dimensionality reduction analyses were done to visualize distinct grouping of MECs from different tissues of interest. Two forms of dimensionality reduction analysis were performed, namely principal component analysis(PCA)—to visualize overall distributions—and T-distributed stochastic neighbor embedding(T-SNE)—to better visualize the clustering of similar groups. Both PCA and T-SNE were performed in R(version 4.2.2)using the M3C package [24].

### Cell culture

Human retinal microvascular endothelial cells(HRMECs; Olaf Pharmaceuticals) and human cardiac microvascular endothelial cells(HCMECs; ScienceCell) were used to validate heterogeneity between carMECs and retMECs *in vitro*. Cells(passage 4–5) were cultured with endothelial cell growth basal medium-2(Lonza) supplemented with microvascular endothelial growth medium-2(Lonza), 10% v/v fetal-bovine serum, and 100μg/ml penicillin/streptomycin in a humidified hood at 37°C with 4% CO_2_. Full growth medium was replaced with serum-free medium once cells reached 80–90% confluency. Cells were incubated in serum-free medium for 18 hours, then treated to normal(NG, 5mM) or high(HG, 25mM) D-glucose concentrations for 48 hours. L-glucose was used as an osmotic control for high glucose treatment.

### RNA isolation, reverse transcription, and quantitative PCR

RNA was extracted from HRMECs and HCMECs using the Sanprep Colum microRNA Mini-Preps Kit(Bio Basic) following the manufacturer’s instructions for total RNA isolation. RNA concentrations were measured using the SpectraMax QuickDrop Spectrophotometer(Molecular Devices). cDNA was synthesized using 2μg of total RNA with the High Capacity cDNA Reverse Transcription kit(Applied Biosystems) according to the manufacturer’s instructions. qPCR was performed using the LightCycler 96 system(Roche Diagnostics). Gene expressions were quantified using the standard curve method and normalized to the housekeeping gene *ACTB*. qPCR data were analyzed and plotted in PRISM(version 10; GraphPad). T-tests were performed to assess the effect of HG in each cell type, and corrections for multiple comparisons were done using the Holm–Šidák method. Adjusted p<0.05 was considered statistically significant.

## Results

### MECs from the retina, kidneys, and heart have distinct basal transcriptomic profiles

At the time of sacrifice, non-diabetic mice weighed 28.00±1.73g, while that of diabetic mice weighed 21.00±1.00g. The non-diabetic mice had average serum glucose levels of 7.73±0.59mmol/L, while the diabetic mice had an average serum glucose of 28.30±4.50mmol/L (none of the mice required insulin intervention during the 2-month period). Diabetic mice further exhibited polyuria, as reflected by the wetness of the bedding, as well as glucosuria and ketonuria as measured via urine strips (S1 Table).

**Fig 2:**
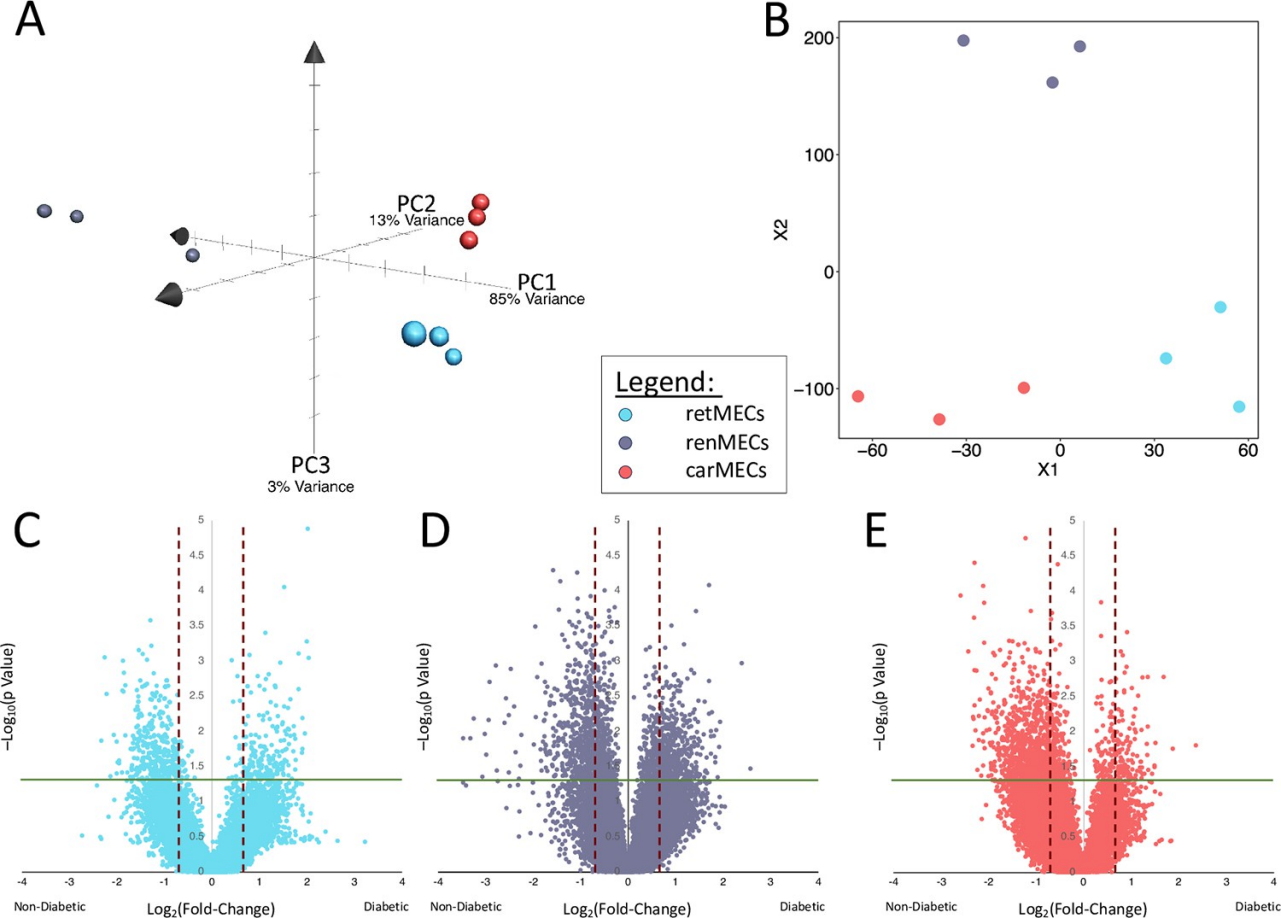
Basal transcriptomic differences across different MECs correspond to differential responses to hyperglycemia. Dimensionality reduction analyses on non-diabetic MECs from the retina, kidney, and heart via A) principal component analysis and B) T-distributed stochastic neighbor embedding. Volcano plots of differentially expressed genes in C) retMECs, D) renMECs and E) carMECs between diabetic and non-diabetic conditions. p values were determined via Student’s T test; n = 3.

We initially compared MECs from different target organs of diabetic complications in non-diabetic to determine whether they are innately distinct as to their transcriptomic profiles. Dimensionality reduction analyses for MECs from non-diabetic mice were performed via PCA and T-SNE. Both showed close grouping of MECs from the same organs of origin, indicating similarity; and more distant grouping between MECs from different organs of origin, indicating dissimilarity (Fig 2A, 2B). Between the different MEC types retinal MECs(retMECs) and cardiac MECs(carMECs) appeared to be closer to each other than either were to renal MECs(renMECs). Both PCA and T-SNE were used for dimensionality reduction analyses because they have different strengths and weaknesses and together provide a better picture of the overall data. PCA is a linear unsupervised approach which better preserves the global structure of the data while T-SNE is a non-linear approach which better preserves local relations between datapoints.

### MECs from different tissues of origin respond differently in diabetes

After demonstrating basal differences between different MEC types, we examined their responses to hyperglycemia. We initially compared diabetic and non-diabetic MECs without separating by tissues of origin. Analysis with pooled MECs from all diabetic vs non-diabetic tissues of interest showed differential expression in 521 genes (S2 Fig). GSEA showed enrichment of Hallmark Pathways(as defined by GSEA, 50 gene sets that summarize and represent well-defined biological states or processes) such as epithelial-mesenchymal transition(a corollary for endothelial-mesenchymal transition in the GSEA analyses [25, 26]), angiogenesis, and various inflammatory pathways in the diabetic group, and enrichment of oxidative phosphorylation and fatty acid metabolism in the non-diabetic group (S2 Fig). Analysis of Canonical Pathways showed upregulation of inflammatory pathways and downregulation of oxidative phosphorylation in diabetes (S2 Fig). Overall, electron transport complex and protein degradation were two of the main clusters of downregulated pathways, while extracellular matrix interactions and PKA activity were upregulated in diabetes (S2 Fig).

**Fig 3:**
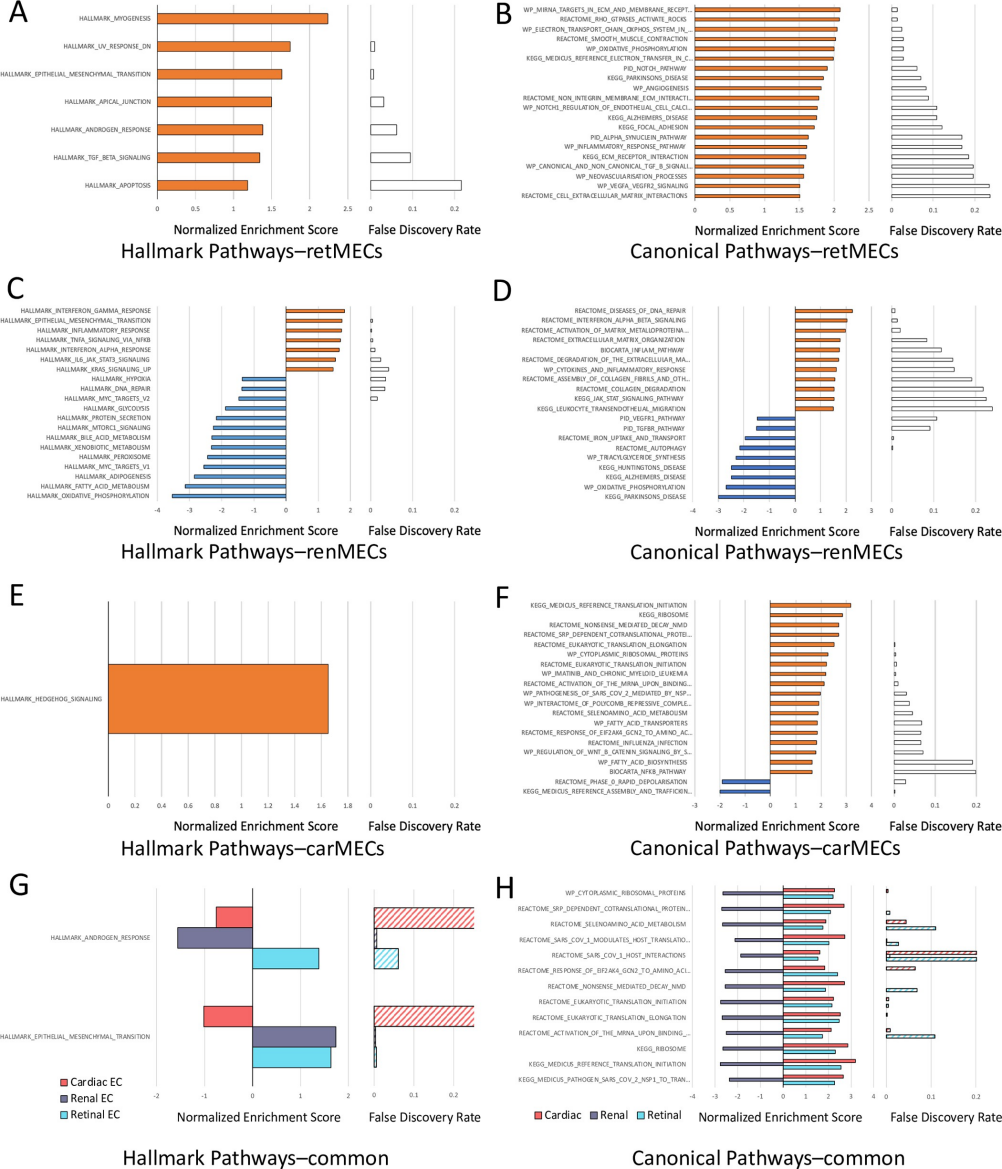
Differential gene set enrichment in retinal, renal, and cardiac MECs in response to hyperglycemia in diabetes. Significantly enriched gene sets from the Hallmark Pathways collection and selected significantly enriched gene sets from the Canonical Pathways collection in retMECs(A, B), renMECs(C, D), and carMECs(E, F). G) Gene sets significantly enriched in two or more MEC types from the Hallmarks Pathways gene set and H) selected significantly enriched gene sets in all MECs from the Canonical Pathways gene set.

Following the initial grouped analysis, we examined the changes in individual MEC types. Volcano plots showed 771 differentially expressed genes in retMECs, 1274 in renMECs, and 1154 in carMECs in response to hyperglycemia in diabetes (Fig 2C–2E); carMECs showed noticeable skew toward the non-diabetic condition (Fig 2E). GSEA showed differential enrichment of Hallmark Pathways across MECs from different organs (Fig 3A, 3C, 3E, 3G). Mesenchymal transition, which was found to be significantly enriched in MECs in diabetes, was only significantly enriched in diabetic retMECs and renMECs, but not in carMECs (Fig 3G). The Hallmark Androgen Response pathway on the other hand, was not found to be altered in grouped MECs, is significantly enriched in retMECs of diabetic mice, enriched in renMECs of non-diabetic mice, and not significantly changed in carMECs (Fig 3G). GSEA revealed the highest number of significantly altered Canonical Pathways in renMECs, while carMECs had the lowest. Enrichment of various inflammatory pathways were present across all MECs (Fig 3B, 3D, 3F). In pathways that were significantly enriched in all three MEC types, retMECs and carMECs often responded similarly to hyperglycemia, while renMECs often responded in the opposite manner (Fig 3H).

### Analysis of selected transcripts show retinal and cardiac MECs respond differently to hyperglycemia *in vitro*

**Fig 4:**
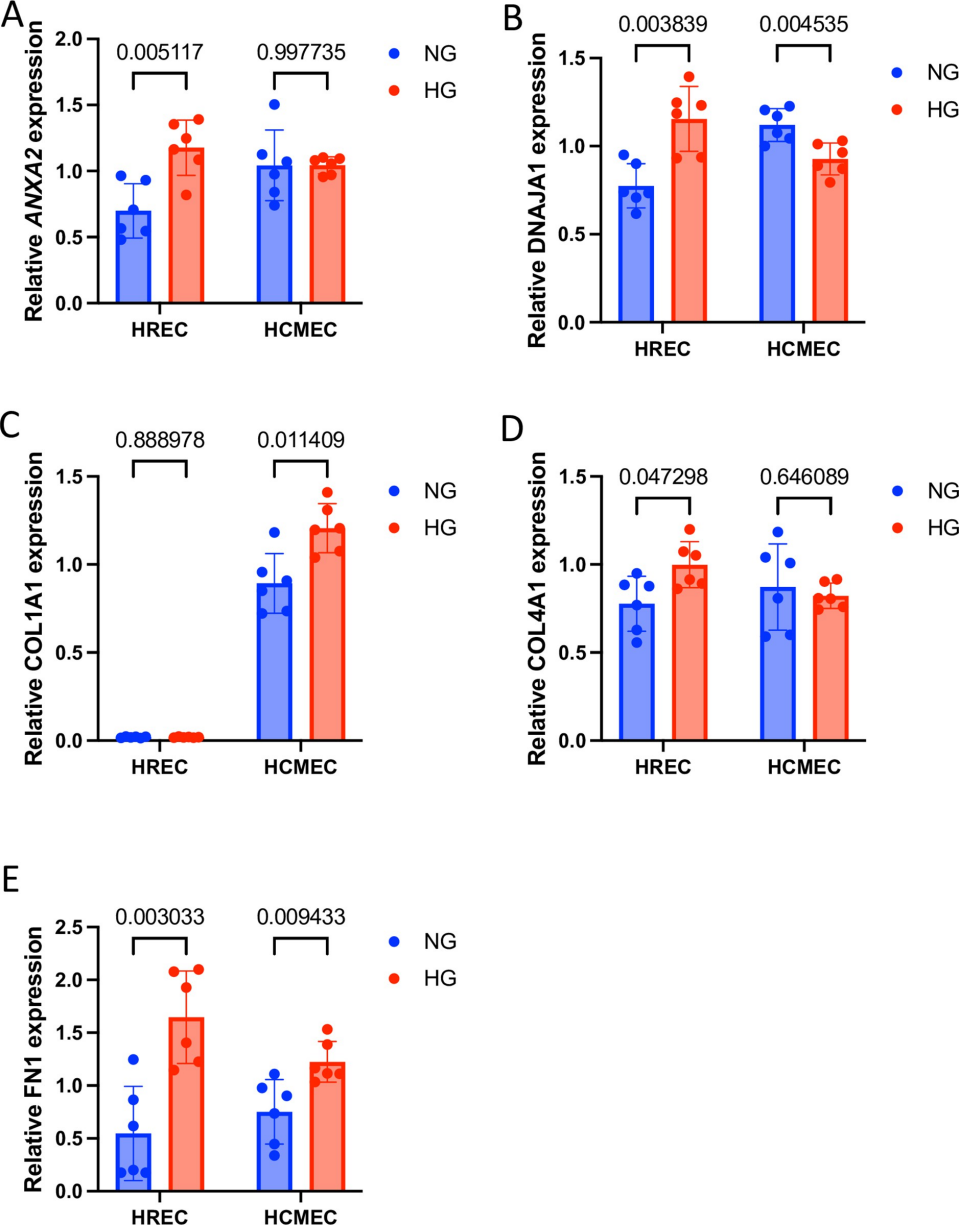
Differential response between retMECs and carMECs to high glucose *in vitro*. High glucose(25 mM; 48 hrs) caused significant regulation of A) *ANXA2* and B) *DNAJA1* in HRMECs. *ANXA2* experienced no significant changes and *DNAJA1* was significantly downregulated in HCMECs cultured with high glucose. HCMECs showed significant upregulation of C) *COL1A1* and no significant alteration of D) *COL4A1* in response to high glucose, while HRMECs showed significant upregulation of *COL4A1* but not *COL1A1*. Both HRMECs and HCMECs showed significant upregulation of E) *FN1*. n = 6 for each group. Gene expressions normalized to *ACTB*. Data expressed as mean ± standard deviation. Adjusted p values < 0.05 were considered significant.

We expanded our study to see if MECs exhibit heterogeneity *in vitro*. To this extent we investigated retMECs and carMECs, using HRMECs and HCMECs respectively, because while they were transcriptomically distinct, they had more in common with one another than with renMECs. MEC-specific changes to *ANXA2* and *DNAJA1*, which were shown by the volcano plots to be significantly upregulated in retMECs, and unchanged and downregulated respectively in carMECs, were reproduced *in vitro*. Both *ANXA2* and *DNAJA1* were significantly upregulated in HRMECs in response to HG, while in HCMECs, *ANXA2* showed no significant changes, and *DNAJA1* was significantly decreased (Fig 4A, 4B). We further examined several key extracellular matrix proteins, because “extracellular matrix interactions” was a notable cluster of gene sets that were shown to be upregulated in GSEA and network clustering analyses. COL1A1 was significantly upregulated in HCMECs in response to HG but showed no significant change HRMECs (Fig 4C). Inversely, COL4A1 was significantly upregulation in HRMECs but not in HCMECs in response to HG (Fig 4D). FN1 was significantly increased in both HRMECs and HCMECs in response to HG (Fig 4E). None of these genes were altered by high osmolarity in either cell type (S3 Fig).

### Tissue factors in the retinal environment do not induce similar responses in retMECs and retinal neuronal cells

**Fig 5:**
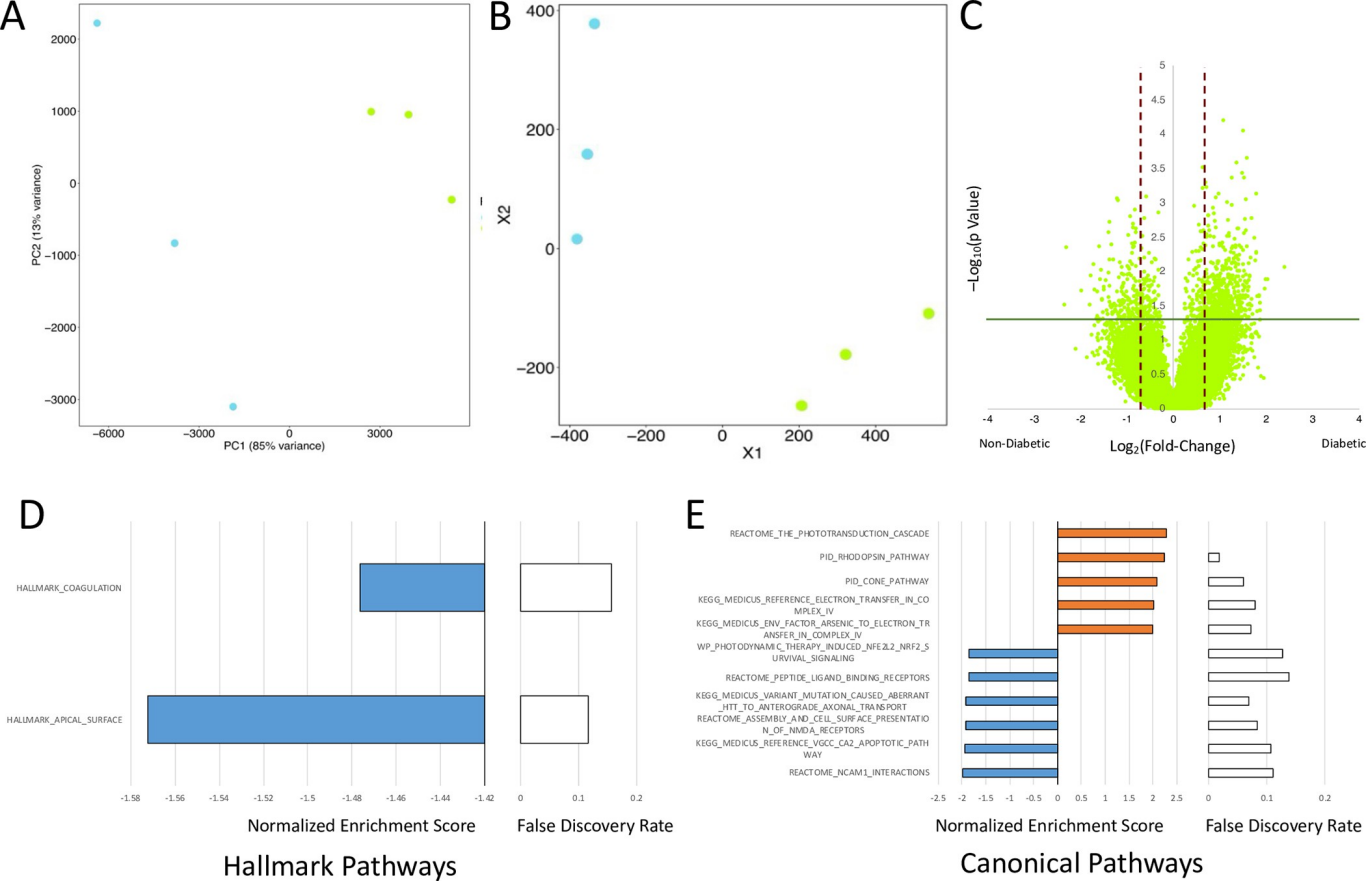
Close integration between retMECs and retinal neuronal cells in the neurovascular unit do not result in similar responses to hyperglycemia in diabetes. Dimensionality reduction analyses on non-diabetic retMECs and retinal neuronal cells via A) principal component analysis and B) T-distributed stochastic neighbor embedding. C) Volcano plot of differentially expressed genes retinal neuronal cells in diabetic vs non-diabetic conditions. p values were determined via Student’s T test; n = 3. Significantly enriched gene sets from D) the Hallmarks Pathways, and E) the Canonical Pathways collections.

We further examined what effects tissue-specific factors had on different cell types situated in the same environment. To this effect, we examined retMECs and retinal neuronal cells. As MECs had been the primary focus of this study, we did not distinguish between subtypes of NeuN^+^ cells of the retina as we had done with CD31^+^ cells. Dimensionality reduction analyses showed that retMECs were different from retinal neuronal cells at the basal level (Fig 5A, 5B). Volcano plot showed 776 differentially expressed genes in neuronal cells (Fig 5C), of which 28 were also significantly changed in retMECs, and only 13 were altered in the same direction. GSEA found enrichment of phototransduction cascade, enrichment of complex IV activity, and decrease in NCAM1 interactions in neuronal cells in response to diabetes (Fig 5D, 5E). No exact matches in significantly enriched pathways were found between retMECs and the neuronal cells, but various pathways relating to electron transport were enriched across the two cell types in response to hyperglycemia.

## Discussion

Hyperglycemia-induced endothelial dysfunction is one of the earliest manifestations of diabetes and is a key process underlying all chronic complications of diabetes [6–8]. In this study, we examined transcriptomic changes in MECs in response to hyperglycemia in early diabetes, both as a broad group, and in separate target organs of common diabetic complications. We focused on early-stage changes in diabetes, as these changes likely constitute the initiating events leading to further tissue damage in chronic diabetic complications. We showed that MECs experience general changes such as downregulation of protein degradation and oxidative phosphorylation and upregulation of extracellular matrix interactions in response to diabetes. However, we found that MECs from different organs largely respond differently to hyperglycemic insult, with most MEC types having more uniquely altered pathways than shared pathways with other MEC types. This is supported by our finding that MECs from different organs are transcriptomically distinct from one another in the healthy state. We further showed that heterogeneity is ingrained in the distinct MEC types, such that HRMECs and HCMECs responded differently to hyperglycemia *in vitro*, out of their original tissue contexts. Separately, we found that despite producing unique responses across MECs, tissue-specific factors may not be sufficient to induce common responses in different cell types within the same organ.

The basic principles of hyperglycemia-induced endothelial damage have long been established. Excessive glucose enters the ECs via the constitutively expressed and insulin-independent glucose transporter, GLUT1, overwhelming the glycolytic and oxidative phosphorylation pathways [6–8]. The glycolytic intermediates get shunted off to more detrimental routes such as the polyol, hexosamine, protein kinase C and AGE pathways [6–8]. The extra flux through the electron transport chain increases ROS generation, which activates antioxidant systems, one side-effect of which is the inhibition a glycolytic enzyme GAPDH [6–8]. These series of intracellular changes occur in all vascular ECs exposed to high circulating glucose. It is therefore no surprise that commonly altered pathways would be produced in MECs of different origins. More interesting is the finding that MECs from different organs have a wider range of unique responses to hyperglycemia, which can be missed when looking at MECs as a general cell population. Gene sets such as WP_NEOVASCULARISATION_PROCESSES, which were only enriched in one type of MEC, and gene sets such as KEGG_ALZHEIMERS_DISEASE which were upregulated in one MEC type, downregulated in another, and not significantly changed in the third, were overlooked when examining MECs as a group. MEC heterogeneity likely reflects the influences by different tissue environments, and differential functions.

Intercellular communication can greatly influence gene expression and cellular behaviour. Transforming growth factor, inflammatory mediators, and angiogenic factors are common signalling factors stimulated by high glucose which induce specific changes in endothelial gene expression [6, 27–29]. However, there are signals that would be differentially present in different tissues depending on the cell types present. For example, retMECs, which are integrated with the neurovascular unit, are affected by hyperglycemia-induced accumulation of the neurotransmitter glutamate in diabetes, which can increase oxidative stress in the retina and retinal ECs [30]. retMECs are also influenced by Müller glia-derived signals, such as microRNA-containing exosomes, which have been reported to modulate angiogenesis [31, 32]. Contrastingly, carMECs are influenced by cardiomyocytes, fibroblasts and specialized conductive cells via paracrine factors [33]. carMECs have also been found to interface with cardiomyocytes via connexins, which form gap junctions, facilitating the passage intracellular signalling molecules [34, 35]. renMECs are surrounded by mesangial cells, podocytes, and the many specialized epithelial cells of the renal filtration system, and studies have highlighted the importance of crosstalk between the various cells in the maintenance of renal function and the progression of diabetic kidney disease [36, 37]. In all, every unique cell type in each tissue introduces a new set of signals which influence and modify the ultimate behavior of the MECs.

Having highlighted the impact that tissue-specific signals have on MEC responses to hyperglycemia, we further examined if common signals would produce similar responses in different cell types in the same tissue. This analysis was focused on the retina, where the MECs are most tightly integrated with non-MECs [10]. Unsurprisingly, retMECs and neuronal cells were different at a basal level. But given the closeness of these two cell types in the retina, retMECs and neuronal cells had unexpectedly little in common in their responses to hyperglycemia in diabetes. Both showed elevation of oxidative phosphorylation-related pathways, but this was the extent of similarities between the two cell types. These findings were surprising to us because signals present in the retinal neurovascular unit, such as increased accumulation of glutamate [30] or Müller glia-derived exosomes [31, 32], should somewhat similarly influence both cell types. One possible explanation for the lack of similar responses may be that the neuropil was less exposed to hyperglycemia in our model. As mentioned, the BRB strictly regulates the passage of substrates into and out of the retina, including glucose [11–13]. Endothelial dysfunction in diabetes eventually compromises the integrity of the BRB, leading to hyperglycemic damage to the rest of the retina [11–13]. Given our model is of early diabetes, it is possible that the neuronal retina did not experience as much hyperglycemic damage compared to retMECs. This would explain why retMECs showed enrichment of inflammatory pathways while the neuronal cells did not, even though inflammation known to occur in the neuronal cells in response to hyperglycemia [31]. Longer-term models may be required to elucidate whether tissue-specific signals produce similar responses in different cell types.

Apart from the differential influence on MECs by non-ECs in different tissues, the innate physiological function of the MEC likely also plays a role in their transcriptomic heterogeneity. retMECs form the BRB, an exceedingly strict barrier. carMECs form “classical” capillaries, supporting the passage of some molecules and nutrients while restricting the movement of larger molecules and fluids [14, 15]. renMECs form leaky fenestrated capillaries, allowing the passage of solutes by simple diffusion [14, 15]. Among the pathways which were significantly enriched in all MEC types in response to diabetes, we found that carMECs and retMECs were consistently more similar to each other and different from renMECs. This finding may be attributable to the fact that renMECs are the odd ones out among the three MEC types, forming fenestrated capillaries, compared to retMECs and carMECs which form continuous capillaries. At the basic level, renMECs lack the tight junctions that help restrict vascular permeability, and in their place, are leaky fenestra that permit free diffusion of solutes [14, 15]. This may inform how renMECs interact differently with, and therefore respond differently to hyperglycemia in diabetes compared to retMECs and carMECs. It must be noted however, that despite being more similar to each other than to renMECs, carMECs and retMECs are still functionally and transcriptomically distinct. Regular continuous capillaries permit limited paracellular transport of glucose while specialized barriers do not [15, 38].

The effects of tissue-specific intercellular signalling and differential functional demands on MEC transcriptomic heterogeneity are persistent, even when MECs are taken out of their tissue contexts. We found differential alteration of a select set of transcripts in response to hyperglycemia in HRMECs and HCMECs. *ANXA2* and *DNAJA1* were genes that were significantly upregulated in HRMECs but showed no change and downregulation respectively in HCMECs. *ANXA2* encodes annexin A2, a protein that can be involved in fibrinolysis, mesenchymal transformation, and collagen secretion [39–42]. *DNAJA1* encodes the DnaJ heat shock protein family(Hsp40) member A1, which has been reported to have angiogenic effects on ECs [43]. Why these genes responded differently to hyperglycemia in HRMECs vs HCMECs would be a matter of speculation, as these would require far more in-depth investigation; however, it is important to note that this pattern of change is consistent between Nanostring and *in vitro* analyses, meaning that MEC heterogeneity persists in the absence of tissue-specific stimuli. We further examined the expressions of several extracellular matrix(ECM) genes, *COL1A1*, *COL4A1*, and *FN1*, because the gene set enrichment analysis indicated enrichment of ECM deposition by MECs in diabetes. *COL1A1* and *COL4A1* encode the alpha 1 subunits of type 1 and type 4 collagen respectively. Type 1 collagen is the predominant type of collagen found in the cardiac ECM [44] and type 4 collagen is the primary type found in retinal ECM [45]. HRMECs showed significant upregulation of *COL4A1*, but not *COL1A1*, whereas HCMECs showed the opposite. Both cell types showed increased expression of *FN1*, which encodes fibronectin, a important ECM protein across both MEC types [46, 47]. This consolidates the notion that MECs exhibit heterogeneity without continual exposure tissue specific factors, and highlights the importance of using tissue-specific ECs for diabetes research.

For this study, we utilized a spatial transcriptomic approach, which provides a novel lens for investigating specific ECs. While we are cognizant that this approach is not without its limitations—such as the limits of resolution for UV cleavage in the Nanostring workflow, or the potential for RNA degradation in FFPE tissues—we believe that the spatial approach offers key benefits and insights. Other approaches, such as single cell sequencing paired with fluorescence-activated cell sorting, often have limited ability to distinguish different types of MECs, for example, intraretinal MECs vs choroidal MECs of the eye, or glomerular MECs vs MECs of the peritubular capillaries of the kidneys. The spatial approach allowed us to selectively analyze specific MECs of interest in our tissues. We further recognize the potential limitations imposed by our usage of male mice, which was a decision based on lack of sex-differences in our previous work done with our early diabetes model. We do, however, understand the importance of using both sexes in cardiovascular research.

In summary, we have shown that MECs from various target organs of diabetic complications show some generic responses to hyperglycemia but are largely distinct from one another, both at the basal level, and in their early responses to diabetes. We further showed that MEC heterogeneity persists outside of their original tissue contexts and may therefore be epigenetically propagated. Separately we investigated the potential of tissue-specific factors driving similar responses in different cell types, and found limited shared responses in retMECs and retinal neuronal cells in our model. While further work may be required to fully elucidate the effect of tissue-specific factors on transcriptomic responses in different MEC types, our current findings highlight the heterogeneity of MECs both in the non-diabetic state and in their responses to hyperglycemic insult. In all, our findings reveal that organ specific MEC heterogeneity may influence the development of pathologic changes in target organs of chronic diabetic complications; such heterogeneity may inform treatment strategies and drug development for different complications.

## Supporting information

S1 Fig Example of identification of regions of interest from retinal, renal, and cardiac tissues. CD31+ regions corresponding to microvascular endothelial cells were identified as regions of interest for UV excision of barcoded oligonucleotides. CD31+ regions corresponding to larger vessels were excluded from UV excision.
(TIF)

S2 Fig Hyperglycemia alters gene expression in MECs. Gene expression data from MECs from retinas, kidneys, and hearts of diabetic or non-diabetic mice (3 each) were pooled and defined as general non-diabetic and diabetic MECs. A) volcano plot of differentially expressed genes between pooled diabetic and non-diabetic MECs. p values were determined via Student’s T test. B) Differentially enriched pathways between pooled diabetic and non-diabetic MECs from the Hallmarks gene set. C) Differentially enriched pathways between pooled diabetic and non-diabetic MECs from the Canonical Pathways gene set. D) Visualization and grouping of differentially enriched pathways from the Canonical Pathways gene set.
(TIF)

S3 Fig Gene expressions in HRECs and HCMECs are not influenced by high osmolarity. Cells cultured with L-glucose (OSM; 25mM; 48 hrs) showed no significance difference in expressions of A) *ANXA2* and B) *DNAJA1* C) *COL1A1* D) *COL4A1* or E) *FN1* compared with NG (5mM; 48 hrs). n = 6 for each group. Gene expressions normalized to *ACTB*. Data expressed as mean ± standard deviation. Adjusted p values < 0.05 were considered significant.
(TIF)

S1 Table Mouse urine glucose and ketone data. (DOCX)

## Abbreviations

BRB: blood-retinal barrier
carMEC: cardiac microvascular endothelial cells
DCM: diabetic cardiomyopathy
DN: diabetic nephropathy
DR: diabetic retinopathy
EC: endothelial cell
ECM: extracellular matrix
HCMEC: human cardiac microvascular endothelial cell
HG: high glucose
HRMEC: human retinal endothelial cell
MEC: microvascular endothelial cel
NG: normal glucose
PCA: principal component analysis
renMEC: renal microvascular endothelial cell
retMECs: retinal microvascular endothelial cell
T-SNE: T: distributed stochastic neighbor embedding
