# Supplementary figures and images for: Differential microvascular endothelial cell responses in the retina in diabetes compared to the heart and kidneys, a spatial transcriptomic analysis
Source: PLoS One. 2024 Dec 31;19(12):e0310949. doi: 10.1371/journal.pone.0310949 (PMC11687817; doi:10.1371/journal.pone.0310949)

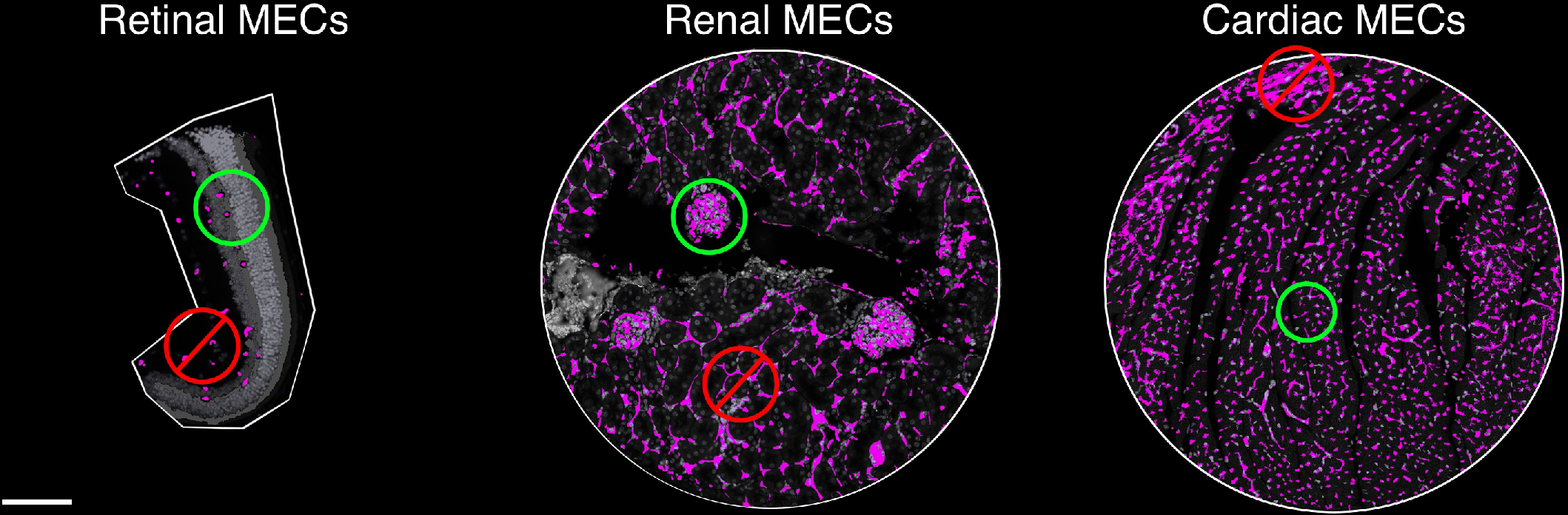

Supplement: S1 Fig — CD31+ regions corresponding to microvascular endothelial cells were identified as regions of interest for UV excision of barcoded oligonucleotides. CD31+ regions corresponding to larger vessels were excluded from UV excision. (TIF) [file pone.0310949.s001.tif]

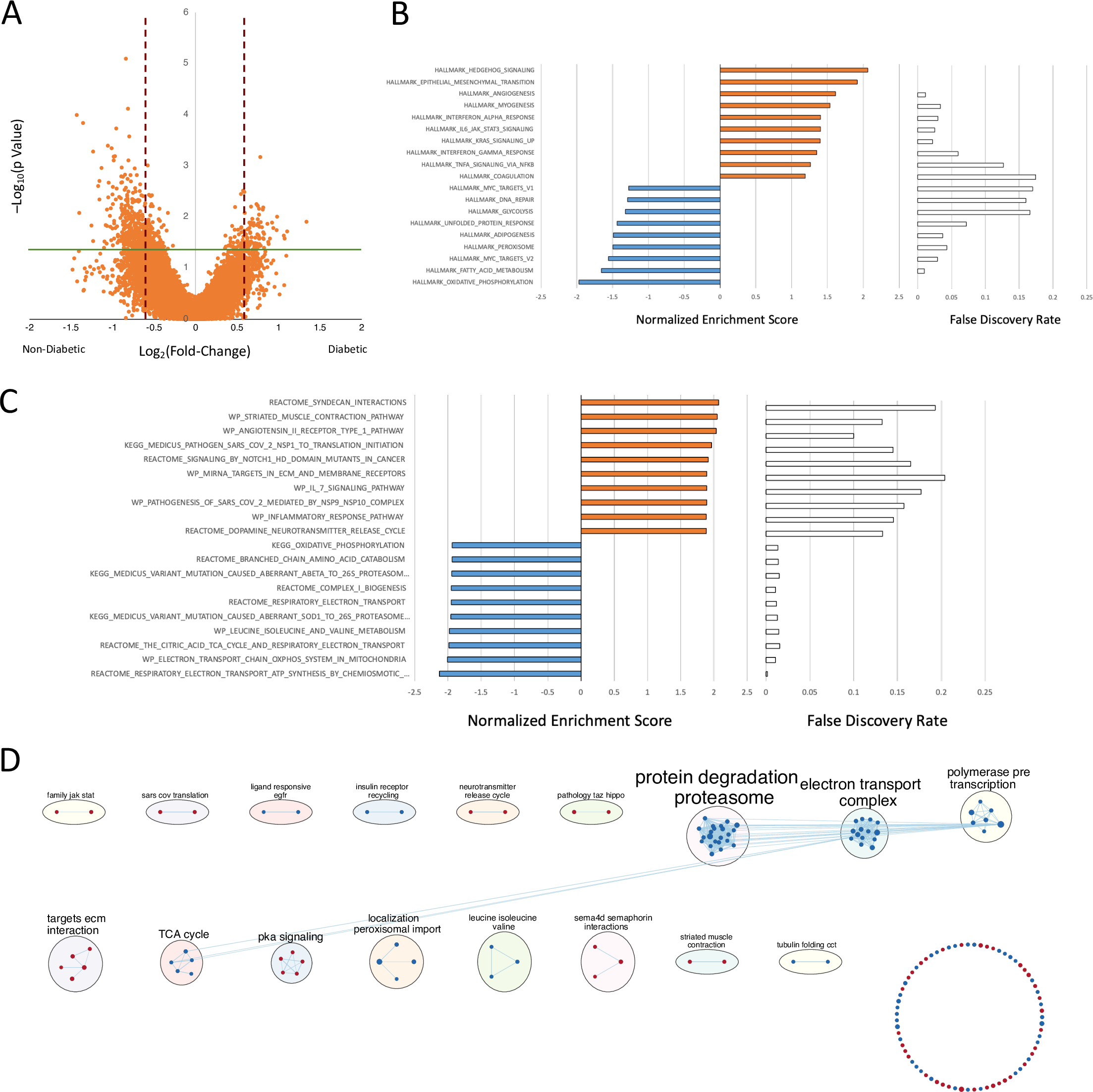

Supplement: S2 Fig — Gene expression data from MECs from retinas, kidneys, and hearts of diabetic or non-diabetic mice (3 each) were pooled and defined as general non-diabetic and diabetic MECs. A) volcano plot of differentially expressed genes between pooled diabetic and non-diabetic MECs. p values were determined via Student’s T test. B) Differentially enriched pathways between pooled diabetic and non-diabetic MECs from the Hallmarks gene set. C) Differentially enriched pathways between pooled diabetic and non-diabetic MECs from the Canonical Pathways gene set. D) Visualization and grouping of differentially enriched pathways from the Canonical Pathways gene set. (TIF) [file pone.0310949.s002.tif]

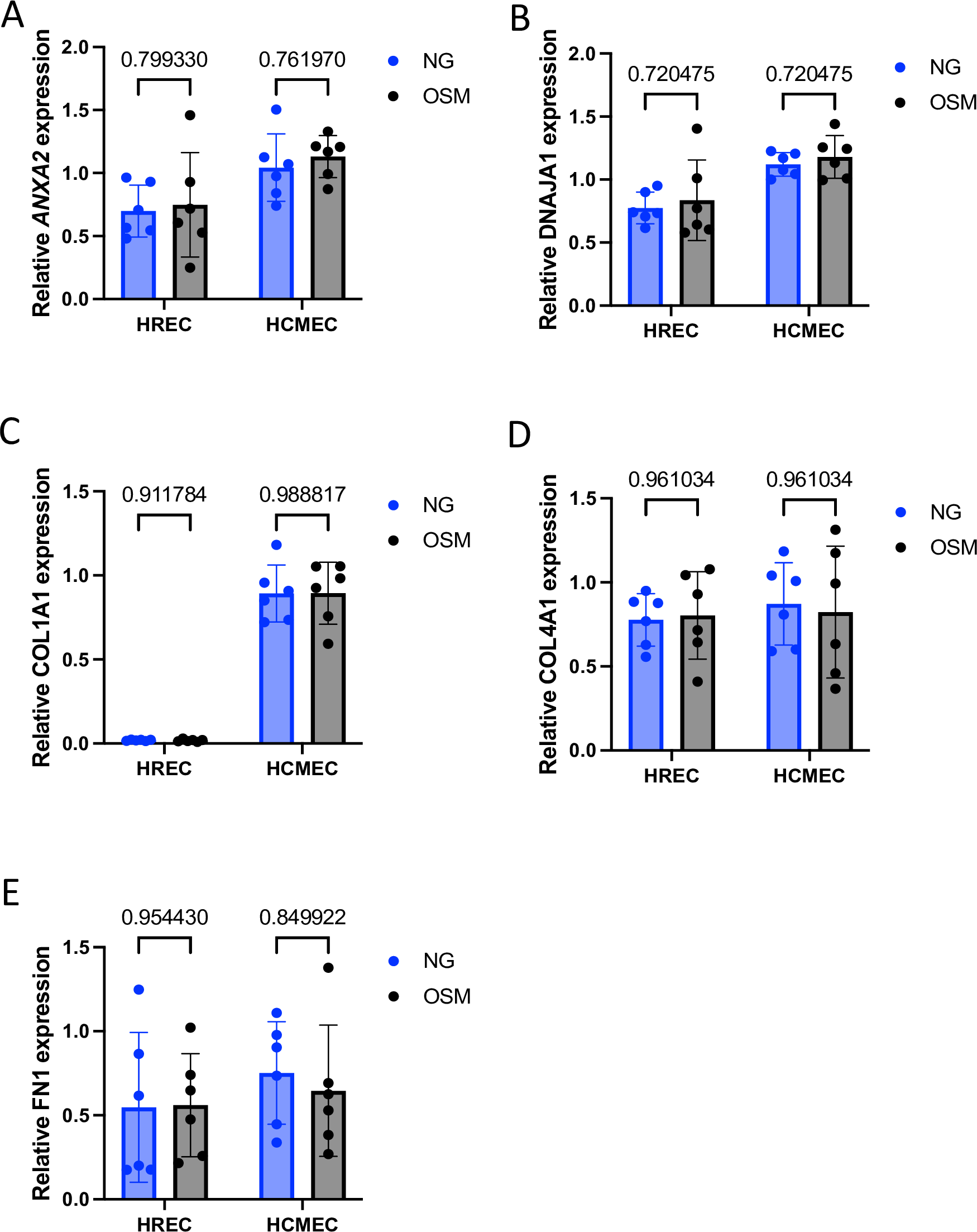

Supplement: S3 Fig — Cells cultured with L-glucose (OSM; 25mM; 48 hrs) showed no significance difference in expressions of A) ANXA2 and B) DNAJA1 C) COL1A1 D) COL4A1 or E) FN1 compared with NG (5mM; 48 hrs). n = 6 for each group. Gene expressions normalized to ACTB. Data expressed as mean ± standard deviation. Adjusted p values < 0.05 were considered significant. (TIF) [file pone.0310949.s003.tif]
