# Supplementary material for: Differential microvascular endothelial cell responses in the retina in diabetes compared to the heart and kidneys, a spatial transcriptomic analysis
Source: PLoS One. 2024 Dec 31;19(12):e0310949. doi: 10.1371/journal.pone.0310949 (PMC11687817; doi:10.1371/journal.pone.0310949)
Supplement: S1 Table — (DOCX) [file pone.0310949.s004.docx]

**Table 1. Mouse urine glucose**

| Animal | | Week | | | | | | | |
| --- | --- | --- | --- | --- | --- | --- | --- | --- | --- |
|  |  | 1 | 2 | 3 | 4 | 5 | 6 | 7 | 8 |
| Non-diabetic | 1 | – | – | – | – | – | – | – | – |
|  | 2 | – | – | – | – | – | – | – | – |
|  | 3 | – | – | – | – | – | – | – | – |
| Diabetic | 1 | – | ± | ± | + | + | + | + | +/++ |
|  | 2 | – | ± | ± | + | + | + | + | +/++ |
|  | 3 | – | ± | ± | ± | + | + | + | + |

Semi-quantitative assessment, as measured by urine strips (URiSCAN GluKeto 2); – = negative, ± = 100 mg/100 mL, + = 250 mg/100mL, ++ = 500 mg/100mL.

**Table 2. Mouse urine ketone**

| Animal | | Week | | | | | | | |
| --- | --- | --- | --- | --- | --- | --- | --- | --- | --- |
|  |  | 1 | 2 | 3 | 4 | 5 | 6 | 7 | 8 |
| Non-diabetic | 1 | – | – | – | – | – | – | – | – |
|  | 2 | – | – | – | – | – | – | – | – |
|  | 3 | – | – | – | – | – | – | – | – |
| Diabetic | 1 | – | – | – | – | – | – | ± | + |
|  | 2 | – | – | – | – | – | – | ± | + |
|  | 3 | – | – | – | – | – | – | – | + |

Semi-quantitative assessment, as measured by urine strips (URiSCAN GluKeto 2); – = negative, ± = 5 mg/100 mL, + = 10 mg/100mL.
